# Homologous recombination deficiency signatures in gastrointestinal and thoracic cancers correlate with platinum therapy duration

**DOI:** 10.1038/s41698-023-00368-x

**Published:** 2023-03-24

**Authors:** Erica S. Tsang, Veronika Csizmok, Laura M. Williamson, Erin Pleasance, James T. Topham, Joanna M. Karasinska, Emma Titmuss, Intan Schrader, Stephen Yip, Basile Tessier-Cloutier, Karen Mungall, Tony Ng, Sophie Sun, Howard J. Lim, Jonathan M. Loree, Janessa Laskin, Marco A. Marra, Steven J. M. Jones, David F. Schaeffer, Daniel J. Renouf

**Affiliations:** 1 Department of Medical Oncology, BC Cancer, Vancouver, BC Canada; 2 Pancreas Centre BC, Vancouver, BC Canada; 3 Canada’s Michael Smith Genome Sciences Centre at BC Cancer, Vancouver, BC Canada; 4 Department of Pathology and Laboratory Medicine, University of British Columbia, Vancouver, BC Canada; 5 Department of Medical Genetics, University of British Columbia, Vancouver, BC Canada; 6 Department of Molecular Biology and Biochemistry, Simon Fraser University, Vancouver, BC Canada

**Keywords:** Cancer genomics, Predictive markers

## Abstract

There is emerging evidence about the predictive role of homologous recombination deficiency (HRD), but this is less defined in gastrointestinal (GI) and thoracic malignancies. We reviewed whole genome (WGS) and transcriptomic (RNA-Seq) data from advanced GI and thoracic cancers in the Personalized OncoGenomics trial (NCT02155621) to evaluate HRD scores and single base substitution (SBS)3, which is associated with *BRCA1/2* mutations and potentially predictive of defective HRD. HRD scores were calculated by sum of loss of heterozygosity, telomeric allelic imbalance, and large-scale state transitions scores. Regression analyses examined the association between HRD and time to progression on platinum (TTPp). We included 223 patients with GI (*n* = 154) or thoracic (*n* = 69) malignancies. TTPp was associated with SBS3 (*p* < 0.01) but not HRD score in patients with GI malignancies, whereas neither was associated with TTPp in thoracic malignancies. Tumors with g*BRCA1/2* mutations and a somatic second alteration exhibited high SBS3 and HRD scores, but these signatures were also present in several tumors with germline but no somatic second alterations, suggesting silencing of the wild-type allele or *BRCA1/2* haploinsufficiency. Biallelic inactivation of an HR gene, including loss of *XRCC2* and *BARD1*, was identified in *BRCA1/2* wild-type HRD tumors and these patients had prolonged response to platinum. Thoracic cases with high HRD score were associated with high *RECQL5* expression (*p* ≤ 0.025), indicating another potential mechanism of HRD. SBS3 was more strongly associated with TTPp in patients with GI malignancies and may be complementary to using HRD and *BRCA* status in identifying patients who benefit from platinum therapy.

## Introduction

There is emerging evidence about the predictive role of homologous recombination deficiency (HRD) in multiple cancers. Much of the focus has centered on *BRCA1/2* and *PALB2* mutations, which have an established function in the HR repair pathway. This has led to large-scale trials demonstrating the efficacy of platinum-based therapies and PARP inhibitors in patients with *BRCA* mutations^1–3^. While *BRCA* and *PALB2* mutations have been recognized as predictive biomarkers in breast and ovarian cancers for some time, their role in gastrointestinal and lung cancers is evolving. The predictive value of germline *BRCA* status was only recently established in pancreatic cancer with maintenance olaparib becoming standard of care, and routine germline testing has now been incorporated into the National Comprehensive Cancer Network (NCCN) guidelines^1,4^. This remains an area of ongoing research in other gastrointestinal cancers^5^. Similarly, retrospective data suggest that *BRCA* and *PALB2* mutations are associated with prolonged responses to platinum-based therapies in lung cancer, although this has yet to be validated in larger prospective studies^6^.

There has been increased recognition of the role of other HRD-related mutations beyond *BRCA* and *PALB2* and their potential to serve as predictive biomarkers. For instance, in patients with HR-deficient pancreatic adenocarcinoma, retrospective analyses support an association between pre-selected HR-deficient mutations and improved survival outcomes with platinum-based treatment^7–9^. The definition of HRD across these studies has been heterogeneous, and largely reliant on commercially available panels^10–12^. It is clear that HRD represents a complicated mechanism, with no single underlying cause, and extends beyond merely a few mutations.

Given the increasingly relevant nature of classification of HRD status in various malignancies, the need to define sensitive and precise methodologies for clinical testing is of significant importance. Selecting patients based solely on a limited number of alterations may exclude a proportion of patients whose tumors still involve the HR pathway^13^. One approach that overcomes these limitations is the measurement of genomic instability and mutation signatures associated with HRD^14^. Several methods for identifying patterns, or signatures, of genomic instability associated with *BRCA1/2* loss have been developed. The HRD score, an aggregation of the loss of heterozygosity (LOH), telomeric allelic imbalance (TAI), and large-scale state transitions (LST), demonstrated high correlation with *BRCA1/2* deficiency and is used as a diagnostic test to provide information on the benefit of PARP inhibitors(i) (myChoiceR CDx test, Myriad Genetics)^15^. A high HRD score has been shown to be predictive of clinical benefit with PARP inhibitor therapy, independent of *BRCA1/2* status in ovarian cancer, but its predictive value of sensitivity to PARP inhibitors or platinum in other tumor types is still unclear^16–20^. SBS3, one of the mutational footprints generated by abnormal double strand break repair, also correlates with *BRCA1/2* deficiency^21^. SBS3 is primarily detected through analysis of whole-genome (WGS) or whole-exome sequencing (WES), but a new bioinformatics tool is being developed to exploit SBS3 from routine cancer gene panels^22^. SBS3 detected from clinical panel sequencing has been shown to be predictive of responses to olaparib in breast and ovarian cancers^23^. Tools such as HRDetect and CHORD that combine multiple genomic signatures based on substitutions, rearrangements or genomic scars determined by WGS show improved accuracy compared to each of the genomic signatures used individually^24,25^. Elevated HRDetect was significantly associated with clinical improvement on platinum-based therapy in advanced breast and pancreatic cancers and with greater sensitivity to rucaparib in primary triple negative breast cancer^26–28^. By surveying the presence of these signatures across human cancers, it is clear that many tumors exhibit HRD-associated signatures even in the absence of *BRCA1/2* mutations. In particular, tumors with loss of function mutations in DNA repair genes, including *PALB2* and *RAD51* paralogs, are associated with HRD-associated signatures, and may similarly respond to platinum-based or PARP inhibitor therapies^25,29–31^.

Whole-genome sequencing readily detects passenger mutations and structural variation associated with HRD. Here we describe the HRD signature landscape derived from whole-genome sequencing (WGS) of gastrointestinal (GI) and thoracic malignancies sequenced as part of the Personalized OncoGenomics program in British Columbia, Canada and characterize the relationship between HRD and clinical outcomes with platinum-based treatment. Retrospective studies to date have suggested an association between platinum response and selected HRD mutations in pancreatic, colorectal, gastric, and lung cancers, although these studies have largely focused on pre-selected HRD mutations rather than a WGS approach^6,8,32,33^. Gastrointestinal and thoracic malignancies were selected for this analysis due to the relative paucity of data in these tumor sites, particularly when compared to breast and ovarian cancers, as well as their routine use of platinum therapies with the potential for HRD to underlie heterogeneity in clinical response^34^.

## Results

### Germline BRCA1/2 mutations in advanced GI and thoracic cancers

**Fig. 1:**
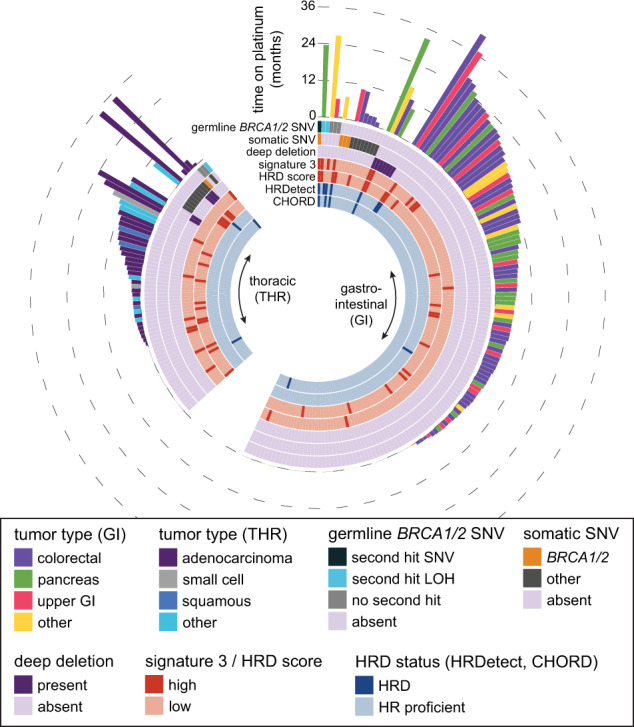
Patient summary. Overview of 223 patients with metastatic gastrointestinal and thoracic malignancies who underwent WGS and RNA-Seq.

We included a total of 223 patients with GI or thoracic primaries in this study (Fig. 1). Of 154 patients with GI primaries, 56% were male and 68% (*N* = 105) were exposed to a platinum agent in the metastatic setting (69% treated with a platinum agent prior to biopsy, Table 1A). Primary sites included colorectal (*N* = 74, 33%), pancreas (*N* = 35, 16%), other GI primary (*N* = 25, 11%), and upper GI (*N* = 20, 9%). Among those with GI cancers, 10 of 154 (6%) cases had a pathogenic or truncating *BRCA1/2* mutation (6 germline, 4 somatic). There were two patients with germline *BRCA2* variants of unknown significance and thirteen patients with a benign *BRCA1/2* alteration (Supplementary Table 1).

**Table 1:** (A) Baseline characteristics and HRD/SBS3 status of patients with advanced gastrointestinal malignancies. (B) Baseline characteristics and HRD/SBS3 status of patients with advanced thoracic malignancies.

**(A) GI cohort**	***N*** = 154
Gender	
Male	87 (57%)
Female	67 (43%)
Tumor site	
Upper GI	20 (13%)
Pancreas	35 (23%)
Colorectal	76 (49%)
Other GI primary	23 (15%)
BRCA 1/2 positive	
Yes	6 germline (4%), 4 somatic (3%)
No	144 (94%)
High HRD score (≥34)	
Yes	20 (13%)
No	134 (87%)
SBS3	
Mean (SD)	(0.03)
High SBS3 (>0.05)	14 (9%)
Somatic mutations in HR genes	
Homozygous deletion	8 (5%)
Heterozygous somatic mutation	9 (6%)
Homozygous somatic mutation	3 (2%)
First-line platinum agent (*N* = 52)	
Cisplatin	18 (35%)
Oxaliplatin	32 (62%)
Carboplatin	2 (4%)
Second-line platinum agent (*N* = 56)	
Cisplatin	10 (18%)
Oxaliplatin	43 (77%)
Carboplatin	3 (5%)
Third-line platinum agent (*N* = 13)	
Cisplatin	2 (15%)
Oxaliplatin	8 (62%)
Carboplatin	3 (23%)
Fourth-line platinum agent (*N* = 7)	
Cisplatin	1 (14%)
Oxaliplatin	6 (86%)
Fifth-line platinum agent (*N* = 2)	
Oxaliplatin	2 (100%)
**(B)** **Lung cohort**	***N*** = 69
Gender	
Male	30 (44%)
Female	39 (56%)
Histology	
Adenocarcinoma	48 (70%)
Squamous cell	5 (7%)
Small cell	2 (3%)
Other thoracic primary	14 (20%)
BRCA 1/2 positive (somatic or germline)	
Yes	3 pathogenic germline (4%), 2 somatic (3%)
No	64 (93%)
High HRD score (≥34)	
Yes	15 (22%)
No	54 (78%)
SBS3	
Mean (SD)	0.009 (0.022)
High SBS3 (>0.05)	8 (12%)
Somatic mutations in HR genes	
Homozygous deletion	5 (7%)
Heterozygous somatic mutation	6 (9%)
Homozygous somatic mutation	2 (3%)
First-line platinum agent (*N* = 43)	
Cisplatin	24 (56%)
Carboplatin	19 (44%)
Second-line platinum agent (*N* = 14)	
Cisplatin	6 (43%)
Carboplatin	8 (57%)
Third-line platinum agent (*N* = 3)	
Carboplatin	3 (100%)
Fourth-line platinum agent (*N* = 3)	
Cisplatin	2 (67%)
Carboplatin	1 (33%)

Of 69 patients with thoracic primaries, 44% were male and 70% (*N* = 48) patients were exposed to platinum-based therapies (67% treated with a platinum agent prior to biopsy, Table 1B). Primary histologies included lung adenocarcinoma (*N* = 48, 22%), lung squamous cell (*N* = 5, 2%), small cell lung (*N* = 2, 1%), and other thoracic primary (*N* = 14, 6%). Among those with thoracic cancers, five of 69 (7%) cases had a *BRCA1/2* mutation (3 germline, 1 somatic, and 1 somatic variant of uncertain significance). There were three patients with benign germline alterations, where one patient had multiple benign mutations. Thirteen of 69 (19%) patients had driver mutations in either *ALK* (*n* = 2) or *EGFR* (*n* = 11). One of the patients with an *EGFR* mutation also had a concurrent somatic *BRCA2* alteration.

### BRCA1/2 mutation signature and HRD score in BRCA1/2 mutant tumors

HRD score, comprised of metrics for loss of heterozygosity, large-scale transitions, and allelic imbalance associated with genomic instability, and COSMIC *BRCA1/2*-associated single base substitution signature 3 (SBS3) are commonly cited measures of HRD in human cancer^20,35^. Given their mutual association with HRD, we examined how well correlated HRD score and SBS3 were in both GI and thoracic cohorts. We detected a significant, but weak positive correlation between these two scores in GI tumors, suggesting that they measure distinct mutational processes that likely correlate with HRD status as well as other tumor features (Pearson correlation coefficient of 0.22, *p* = 0.01). In contrast to GI malignancies, there was no correlation between HRD score and SBS3 exposures in the thoracic cohort (Pearson correlation coefficient of 0.02, *p* = 0.86).

Six GI patients had both high HRD score and high SBS3 exposure, with three pathogenic germline *BRCA1/2* mutant patients falling into this group (Table 2). A second somatic hit was detected in two of the *BRCA1/2* positive cases, with a single-nucleotide variant resulting in a frameshift *BRCA2* mutation in one sample and a loss of heterozygosity in the other sample. In the third germline sample with a pathogenic *BRCA2* mutation, no somatic alterations in *BRCA1/2* or in other HR genes were observed and we did not find any evidence of epigenetic silencing of *BRCA1/2* since both genes showed high expression compared to TCGA tumors. The three additional GI tumors with pathogenic germline *BRCA1/2* mutations had a low HRD score and only one of them had high SBS3 exposure. Among the thoracic cancer cohort, only one patient exhibited concurrent high HRD score and SBS3, and this patient harbored a *BRCA2* mutation that was classified as benign (T582P, clinvar variant accession VCV000037753.10) (Table 2 and Fig. 1). Fifteen patients (22%) with thoracic tumors had a high HRD score and eight (12%) had a high SBS3 exposure. Interestingly, none of the patients with a pathogenic *BRCA1/2* germline mutation had high HRD score and only one patient with biallelic inactivation had high SBS3 exposure (Fig. 1). Since some thoracic malignancies carry an increased mutational load secondary to smoking that might negatively impact the detection of SBS3, SBS3 exposures were further examined in thoracic tumors with and without smoking signatures (SBS4). No mutations associated with SBS3 were observed in malignancies with SBS4. Microhomology deletions, which are also strongly associated with HRD, were absent in most malignancies with smoking signatures, indicating the lack of HRD tumors in the smoking cohort (Supplementary Fig. 1). Two samples with smoking signatures, however, had a high proportion of microhomology deletions, but no SBS3-associated mutations; therefore it is possible that the detection of SBS3 is limited by the high number of smoking-associated mutations.

**Table 2:** DNA repair mutations among patients with both high HRD and high SBS3 scores. For tumors that were classified as HRD by HRDetect or CHORD, the scores are indicated.

Patient	Tumor site	HRD score	SBS3	HRDetect	CHORD	Germline HR mutations (second hit)	Somatic HR mutations
1	GI	52	0.223	0.999	0.794	BRCA2 (somatic snv)	BRCA2 (p.Q2384fs), BLM (p.L325fs)
2	GI	43	0.0534	0.814	–	–	RIF1 (p.S2049A)
3	GI	40	0.126	0.996	0.814	BRCA2 (none)	–
4	GI	38	0.0722	–	–	BRCA1 (LOH)	–
5	GI	36	0.0782	–	–	–	RAD51B homdel
6	Thoracic	36	0.0688	–	–	BRCA2^a^(LOH)	SLX4 (p.C336G)
7	GI	35	0.0827	0.986	0.522	–	XRCC2 homdel

^a^Benign (T582P), het in germline, hom in tumor.

Somatic mutations affecting *BRCA1/2* without germline alteration were identified in six of 223 patients (four thoracic and two GI), where four were truncating or expected to be deleterious or pathogenic. Of the cases with somatic *BRCA1/2* mutations, none of them had concurrent high HRD score and high SBS3 exposure, but in all cases the mutations affected only one copy of the gene suggesting a lower impact of monoallelic somatic events on HRD.

### BRCA1/2 mutation signature and HRD score in BRCA1/2 wild-type tumor

**Fig. 2:**
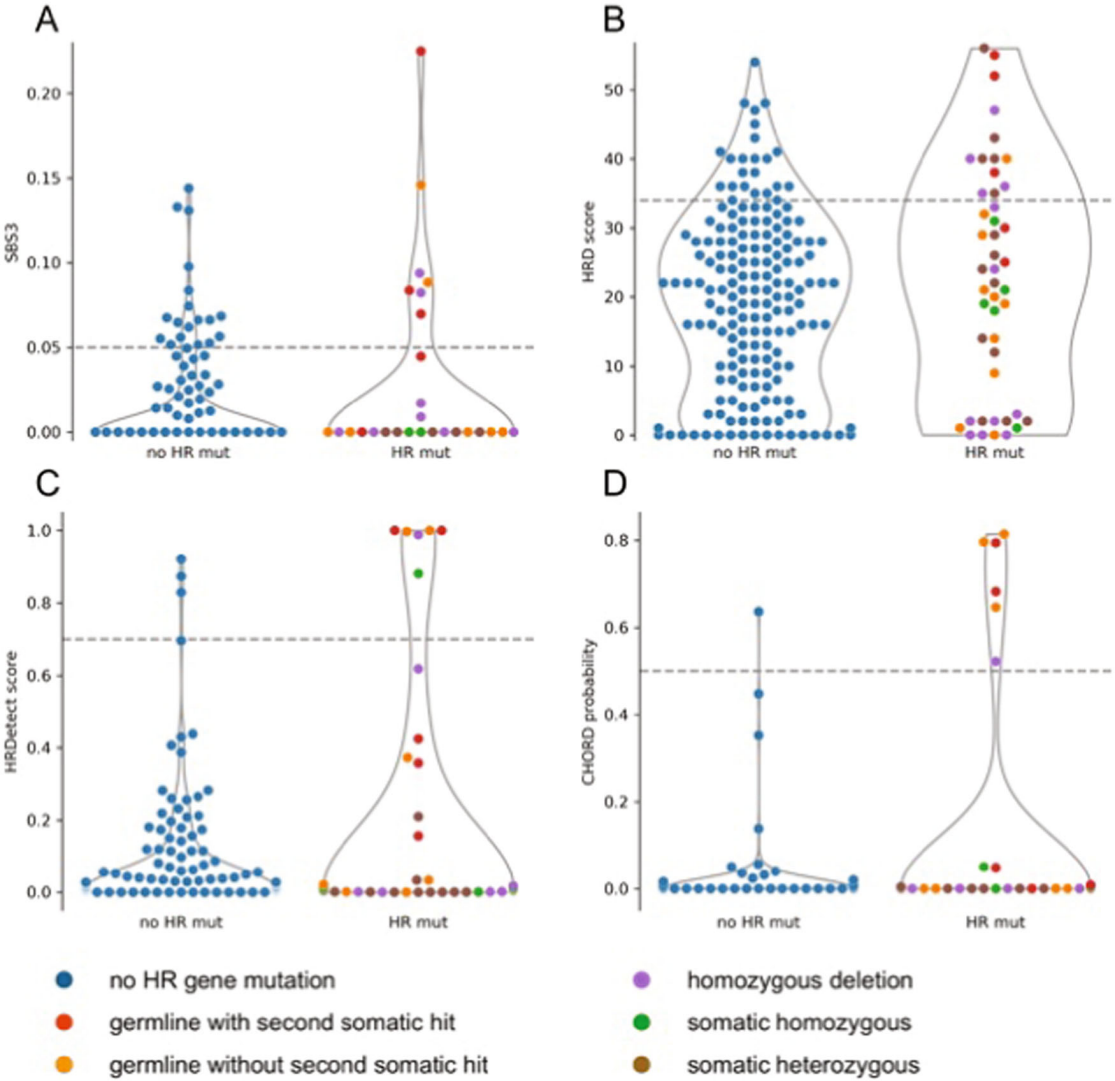
Violin plot of HRD metrics (SBS3, HRD score, HRDetect and CHORD) with pathogenic HR gene alterations. SBS3 (**A**), HRD score (**B**), HRDetect score (**C**) and CHORD probability (**D**) depicted in the combined gastrointestinal and thoracic cohorts with or without pathogenic or deleterious mutations or homozygous deletions in the 51 HR genes listed in the “Methods”. Deleterious mutations included nonsense and frameshift mutations. Thresholds as indicated by the dashed lines are 0.05 (SBS3), 34 (HRD score), 0.7 (HRDetect score) and 0.5 (CHORD probability).

High HRD and SBS3 scores were co-occurring in three of six patients that lacked *BRCA1/2* mutations (all three with GI malignancies). We surveyed a panel of 51 HR genes (see Methods) for mutations that contribute to HRD in the absence of a *BRCA1/2* mutation. Pathogenic or deleterious somatic mutations or homozygous deletions of HR genes are uncommon events in both GI (12%) and thoracic (18%) malignancies, but can contribute to high HRD score and SBS3 (Fig. 2). Whole-genome sequencing revealed a homozygous deletion of the RAD51 paralog, *XRCC2*, in one of the *BRCA1/2* negative samples with high HRD and SBS3, also described by Golan et al.^27^ Transcriptomic analysis confirmed the loss of *XRCC2*, since low RNA expression was detected when compared to disease TCGA datasets (9th percentile). Deletion of two of the three copies of another RAD51 paralog, *XRCC3*, was also observed in this sample. RAD51 paralogs function with RAD51 to promote strand invasion and play a critical role in homologous recombination repair; therefore, the losses of *XRCC2* and *XRCC3* in this patient likely contributed to the HRD phenotype. A homozygous deletion affecting another RAD51 paralog, *RAD51B*, was detected in another *BRCA1/2* negative sample with high HRD score and SBS3, suggesting that HRD in the gastrointestinal cohort might be the result of loss of the RAD51 family members in the absence of *BRCA1/2* alterations. No truncating DNA alterations were found in the third *BRCA1/2* negative sample with high HRD and SBS3 (Patient 2 in Table 2); however, a missense mutation with unknown significance in *RIF1* and low RNA expression of several HR genes, including *BRCA1* (6th percentile), *BRIP1* (6th percentile), *RAD51* (0 percentile) and *RAD51B* (5th percentile) were observed when compared to the TCGA COAD and READ datasets.

Across the entire cohort, 28 of 35 high HRD score tumors lacked high SBS3, and 14 of 21 high SBS3 tumors lacked high HRD scores. Three patients with high SBS3 exposures and low HRD scores had germline *BRCA1/2* mutations, two of which were pathogenic. One patient with lung adenocarcinoma and pathogenic germline *BRCA2* mutation had biallelic gene inactivation due to the loss of heterozygosity, but did not receive platinum-based therapy. Another patient with high SBS3 and a low HRD score had a benign germline *BRCA2* mutation, which was homozygous in the tumor due to loss of heterozygosity. This patient with high-grade neuroendocrine carcinoma of the rectum did not receive platinum-based therapy. The third patient with a pathogenic germline *BRCA1* mutation and anal squamous cell carcinoma did not possess a somatic *BRCA* alteration, but the *BRCA1* RNA expression was low (16th percentile) in the tumor, suggesting a potential silencing of the wild-type allele by epigenetic mechanisms such as methylation. WGS also revealed a potentially disruptive *FANCC* translocation event in this tumor. Deficiency in homologous recombination repair has been described in cells with defects in Fanconi anemia proteins including FANCC; thus, a defect in *FANCC* may have contributed to the HRD phenotype in this patient^36^. This patient had an initial response to platinum (first-line carboplatin, then second-line cisplatin), but later was found to have radiographic disease progression after 26.8 months.

The remaining eleven of 14 patients with high SBS3 and low HRD score had no germline or somatic *BRCA1/2* mutations. One of these patients had gastric adenocarcinoma, and was found to have somatic alterations in *RIF1* and *HELQ*, both of which are genes known to be involved in the HR pathway^37,38^. All of these alterations are variants with unknown significance and the patient had a high tumor mutation burden, suggesting that these alterations might in fact be passenger mutations. Upon review of the RAD51 paralogs, both *RAD51B* (7th percentile) and *RAD51D* (6th percentile) showed low RNA expression in the tumor compared to the TCGA STAD dataset. Despite an initial response to first-line cisplatin and capecitabine, this patient later demonstrated disease progression after 7.2 months.

The other patients did not have somatic alterations within the DNA repair pathway, but low RNA expression of the RAD51 paralogs, *RAD51C* (2nd percentile), *RAD51D* (7th percentile) and *XRCC3* (0 percentile) was observed in one case. The epigenetic silencing of RAD51C via promoter hypermethylation has been shown to correlate with HRD in ovarian cancer^39,40^. The absence of driver mutations in these cases suggests that the high SBS3 score may also identify patients with HRD mediated by a different mechanism from the established HR genes^41,42^.

### RECQL5 expression in patients with high HRD score

**Fig. 3:**
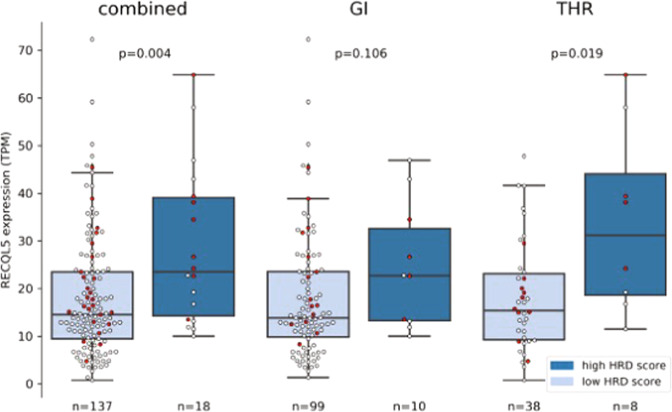
Box plot showing RECQL5 expression in patients with low and high HRD scores in combined and individual GI and thoracic (THR) cohorts. The data points are colored in red if the *RECQL5* gene is subjected to copy gain or amplification. All *p* values presented on the boxplots are determined by Wilcoxon rank sum test (Holm-Bonferroni correction). Box plots represent the median (black line), upper (75th) and lower (25th) quartiles of the distribution and whiskers represent the limits of the distribution (1.5-times interquartile range).

Many of the tumors with high HRD or high SBS3 exposures lacked mutations in HR genes. We investigated the expression of HR genes among patients who had either high HRD score or SBS3 exposure without germline or somatic HR gene alterations to identify potential alternate mechanisms underlying the observed signatures. Out of the 51 examined genes known to be involved in HR (see Methods), the expression of *RECQL5* showed significant association with high HRD score but not with SBS3 exposure in our cohort (Fig. 3 and Supplementary Fig. 2A). After adjusting for tumor type, only thoracic cases with high HRD score retained significance with high *RECQL5* expression (*p* = 0.0189). RECQL5 expression was higher in cases with multiple copy gains, supporting the hypothesis that RECQL5 activity may be elevated in cases with high RECQL5 expression (Fig. 3 and Supplementary Fig. 2B). *RECQL5* plays a role in DNA single and double strand break repair, and functions to limit the formation of RAD51 filaments required for canonical homology directed repair^43^. Increased expression of RECQL5 has been shown to inhibit HR, and promote mutagenic end joining, suggesting increased RECQL5 may phenocopy *BRCA1/2* tumors. Given the important regulatory role of *RECQL5* in HR, the increased levels of *RECQL5* may disrupt canonical repair at double strand breaks as was shown in tumors with *RECQL5* amplification and increased expression and contribute to the high HRD score observed in these patients^44^. Elevated RECQL5 expression, however, was not significantly associated with longer platinum treatment (*p* = 0.195) duration in our cohort suggesting an alternative mechanism that might not lead to enhanced platinum sensitivity.

### Association of BRCA1/2 signatures with survival

**Fig. 4:**
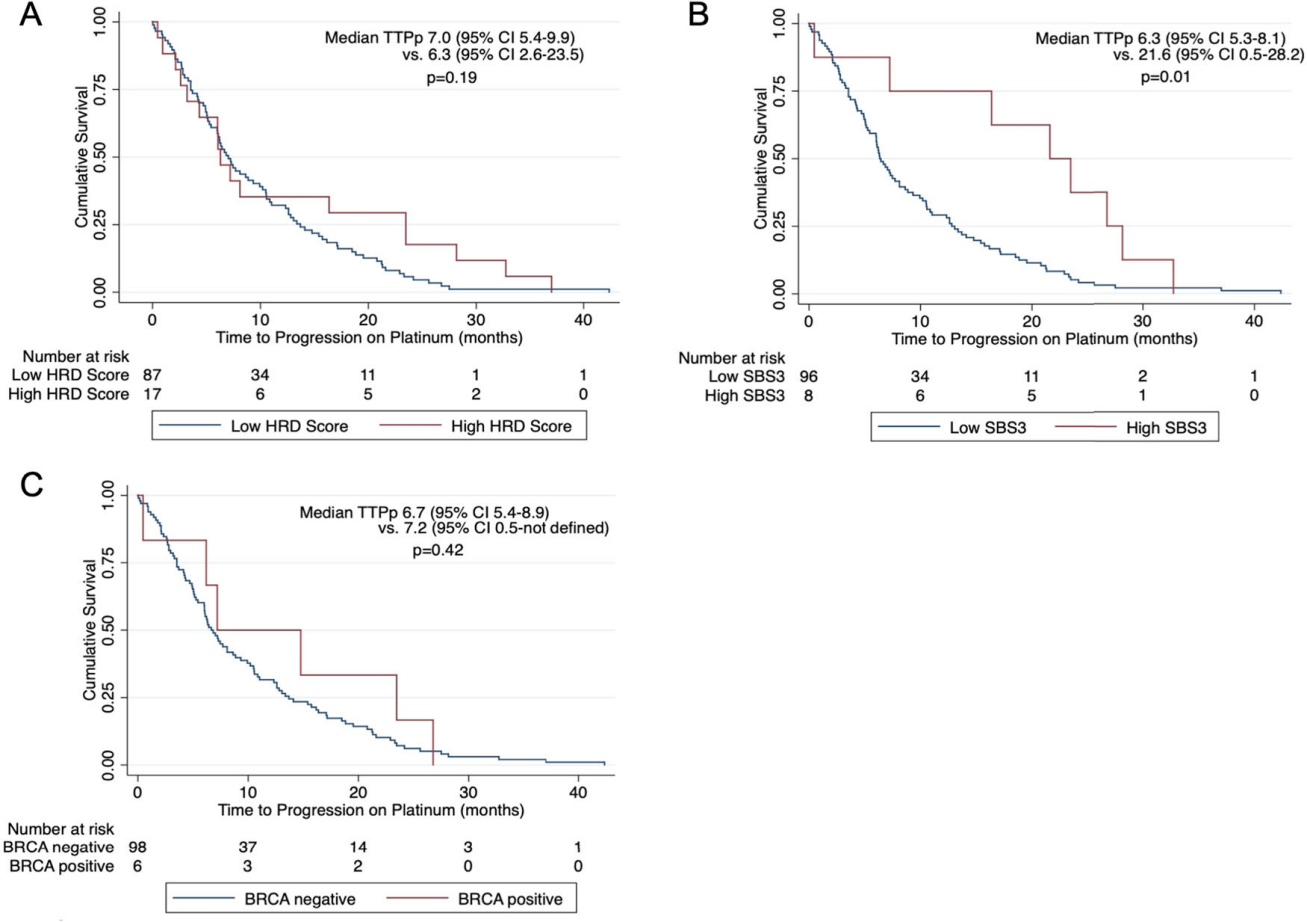
Kaplan–Meier time to progression on platinum curves for patients with metastatic gastrointestinal malignancies. Curves are stratified by HRD score (**A**), SBS3 exposure (**B**), and BRCA status (**C**).

We sought to evaluate whether SBS3 or HRD score were predictive of response to platinum-based therapy in GI malignancies. Kaplan-Meier survival analysis demonstrated that time to progression on platinum therapy was independently associated with SBS3 (*p* = 0.01), but not HRD score (*p* = 0.19) or *BRCA1/2* mutation status (*p* = 0.42; Fig. 4). With Cox regression analysis, SBS3 remained significantly associated with longer time to progression on platinum therapy (HR 0.35, 95% CI 0.14–0.91, *p* = 0.03), while adjusting for tumor site and number of lines of platinum-based therapy (Supplementary Table 2). HRD score and *BRCA* mutation status were not associated with longer TTPp on Cox regression analysis when adjusting for the same covariates (*p* = 0.99, *p* = 0.29, respectively). There was no difference in OS when stratified by HRD score (*p* = 0.86), *BRCA* status (*p* = 0.94), or SBS3 score (*p* = 0.33; Supplementary Fig. 3). On Cox regression analysis, longer OS was associated with increased duration of platinum-based therapy (HR 0.98, 95% CI 0.95–0.99, *p* = 0.04), while adjusting for tumor site, HRD and SBS3 scores, and *BRCA1/2* mutation status. Type of platinum agent was also not prognostic.

**Fig. 5:**
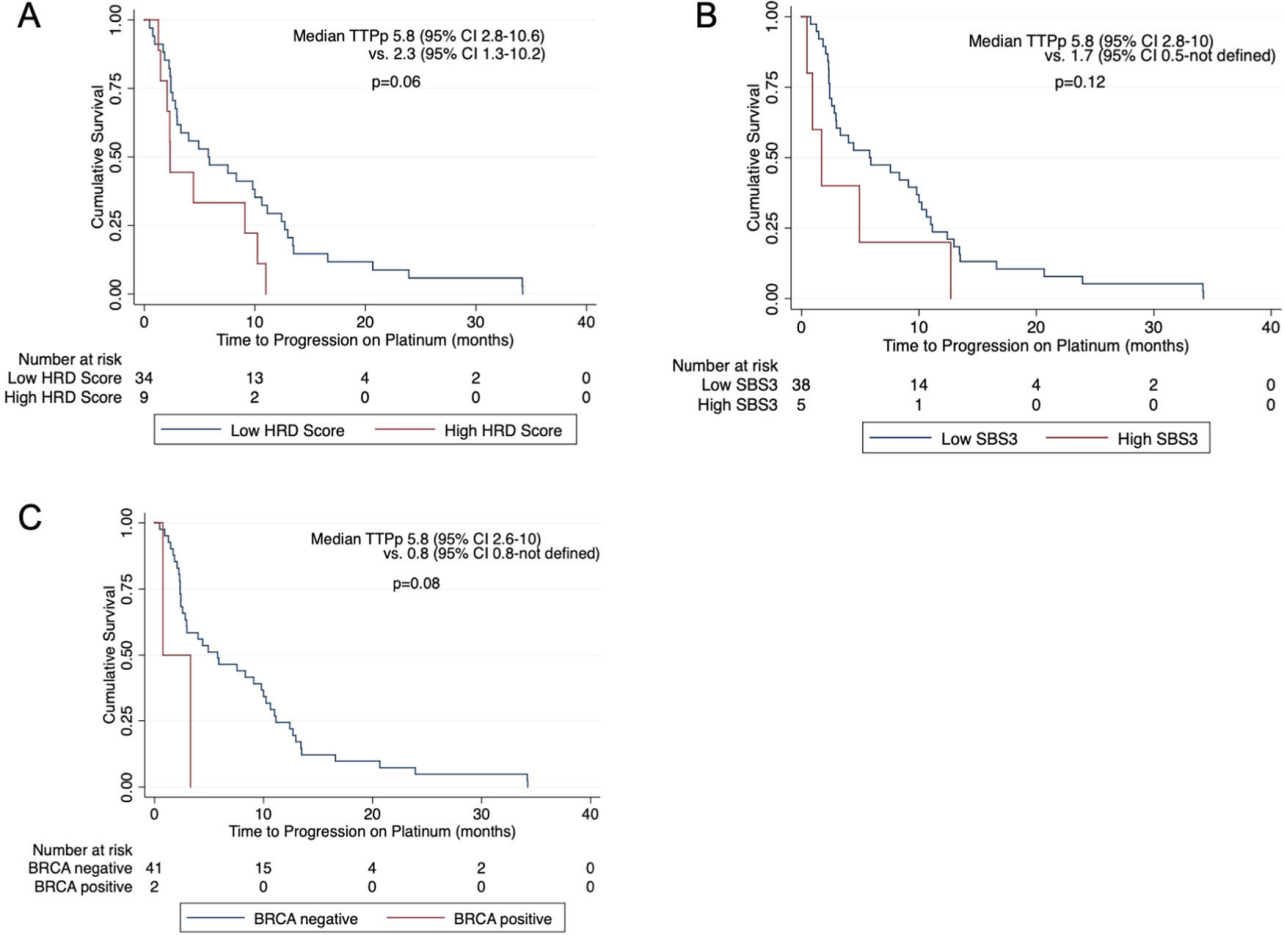
Kaplan–Meier time to progression on platinum curves for patients with metastatic thoracic malignancies. Curves are stratified by HRD score (**A**), SBS3 exposure (**B**), and BRCA status (**C**).

There was no association between HRD score (*p* = 0.06) or SBS3 exposure (*p* = 0.12; Fig. 5) with time to progression on platinum on Kaplan-Meier survival analysis in the thoracic cohort. There was also no significant difference in OS on Kaplan-Meier analysis when stratified by HRD (*p* = 0.49) or SBS3 (*p* = 0.06; Supplementary Fig. 4A and B) scores. We sought to similarly evaluate the TTPp and OS in the thoracic cohort. Interestingly, the five patients with *BRCA1/2* mutations had shorter median OS, measuring 11.7 months compared to 46.3 months (*p* < 0.01; Supplementary Fig. 4C). Two of the patients with *BRCA* mutations received platinum-based treatment. The other three patients had known driver mutations (2 *EGFR* mutations, 1 *ALK* mutation).

### HRD classifiers: HRDetect and CHORD

HRDetect, which aggregates different mutational signatures including single base substitution signatures, structural variant signatures and microhomology-mediated deletions, was shown to predict platinum sensitivity in breast and pancreatic cancers^26,27^. Classifier of Homologous Recombination Deficiency (CHORD), a random forest classifier, is another tool shown to accurately predict HRD across cancer types and discriminate *BRCA1* and *BRCA2* subtypes^25^. Evaluation of HRD in this cohort using both HRDetect and CHORD revealed eight patients in the GI cohort and one from the thoracic cohort as HRD by HRDetect (HRDetect >0.7) (Table 3), and five patients from the GI cohort and two from the thoracic cohort as HRD by CHORD (Table 4). HRD and CHORD each identified 44% of patients with pathogenic germline *BRCA1/2* alterations (Tables 3 and 4). Three of these four patients were identified as HRD by both HRDetect and CHORD. We also noted that only four of the patients with HRD status by HRDetect were identified as HRD by CHORD, suggesting that certain signatures were more strongly associated with HRD in HRDetect than in CHORD.

**Table 3:** HR mutations among patients with HRD as identified by HRDetect.

Patient	Tumor site	HRD score	SBS3	Germline HR mutations (second hit)	Somatic HR mutations
1	GI	52	0.223	BRCA2 (somatic snv)	BRCA2 (p.Q2384fs), BLM (p.L325fs)
2	GI	43	0.0534	–	RIF1 (p.S2049A)
3	GI	40	0.126	BRCA2 (none)	–
7	GI	35	0.0827	–	XRCC2 homdel
8	GI	9	0.0806	BRCA1 (none)	–
10	GI	48	0.0355	ATM^a^ (none)	RBBP8 (p.K537M)
11	GI	30	0.037	BRCA1 (LOH)	–
12	GI	0	0.0	BRCA1^a^ (none)	–
13	Thoracic	31	0.0	–	BARD1 (p.G320^a^)

Note: Patient numbers are continued from Table 2 for continuity.

^a^Benign.

**Table 4:** HR mutations among patients with HRD as identified by CHORD.

Patient	Tumor site	HRD score	SBS3	Germline HR mutations (second hit)	Somatic HR mutations	Subtype
1	GI	52	0.223	BRCA2 (somatic snv)	BRCA2 (p.Q2384fs), BLM (p.L325fs)	BRCA2
3	GI	40	0.126	BRCA2 (none)	–	BRCA1
7	GI	35	0.0827	–	XRCC2 homdel	BRCA2
14	GI	9	0.0806	BRCA1 (none)	–	BRCA1
15	GI	1	0.0	ATM (none)	–	BRCA2
16	Thoracic	1	0.0	–	–	BRCA2
17	Thoracic	25	0.0608	BRCA2 (LOH)	RAD51D (p.T97I)	BRCA2

Note: Patient numbers are continued from Tables 2 and 3 for continuity.

Notably, the patient with the *XRCC2* homozygous deletion and *XRCC3* copy losses, similar to *BRCA1/2* patients, exhibited a high HRDetect score (0.988) and was classified as HRD by CHORD.

A patient with lung adenocarcinoma and with a homozygous stop-gain mutation in BARD1 (G320*) and a duplication event in *RBBP8* (e3–4 duplication) was also classified as HRD by HRDetect but not by CHORD. *BARD1* is a breast cancer susceptibility gene and deleterious variants were shown to be deficient in homologous recombination repair, implying that the truncating BARD1 that lacks several C-terminal functional domains contributed to the HRD phenotype^45^. Among patients with breast cancer, *BARD1* has been associated with HRD tumors although this has not been reported in other tumor types^46^. This patient demonstrated a prolonged response to first-line carboplatin and gemcitabine, with time to progression of 34.2 months.

Three patients with high HRDetect (cholangiocarcinoma, colorectal cancer, stomach adenocarcinoma) did not have pathogenic mutations in HR genes, although the whole-genome sequencing data revealed a benign homozygous germline mutation in *ATM* and a somatic variant of unknown significance in *RBBP8* in the patient with intrahepatic cholangiocarcinoma and a benign germline mutation in *BRCA1* in the patient with stomach adenocarcinoma. Two of these patients demonstrated a prolonged response to platinum, measuring 37.03 and 28.17 months, respectively, suggesting that mutational signatures might be used to predict platinum response in the absence of HR gene alterations.

Within the GI cohort, there was no difference in OS with Kaplan–Meier survival analysis when stratified by HRDetect (*p* = 0.84) or CHORD (*p* = 0.97). With Cox regression, HRDetect was associated with longer time to progression on platinum (HR 0.34, 95% CI 0.12–0.99; *p* = 0.048), however CHORD was not (HR 0.44, 95% CI 0.11–1.83; *p* = 0.26), although cautious interpretation of these observations is warranted given the limited number of patients with positive HRD status by HRDetect and CHORD. There were too few patients identified by HRDetect and CHORD in the thoracic cohort for a meaningful regression analysis.

Among this cohort of patients with GI and thoracic malignancies who underwent detailed molecular profiling with WGS/RNA-Seq, 55% (five of nine) of pathogenic germline *BRCA1/2* cases were predicted as HRD by SBS3, 33% (three of nine) by HRD score and 22% (2 of 9) by both HRD score and SBS3. 44% (4 of 9) were identified as HRD using HRDetect and CHORD.

## Discussion

Here we describe the landscape of mutation signatures associated with HRD in a cohort of advanced GI and thoracic cancers, and explore the association with known and novel mechanisms of HRD. SBS3 and HRD scores have been associated with both germline and somatic defects in *BRCA1/2* across several tumor types^47^. High SBS3 and high HRD score were detected in several tumors with no alterations in HR genes, while aggregation of several HRD-associated signatures into a probability score using HRDetect or CHORD is more strongly associated with mutations in HR genes. Mutational SBS3 and HRDetect were associated with TTPp in patients with GI malignancies, adding to growing evidence that HRD-associated mutational signatures may be beneficial in predicting response to platinum-based therapy. Our data highlight that alternate mechanisms of HRD may in fact increase the currently reported prevalence of HRD in GI and thoracic malignancies. Inclusion of these markers in prospective studies of GI tumors in particular will be paramount in testing this hypothesis.

The mechanism of HRD is complex, as reflected by the variable definitions between studies. *BRCA1/2* alterations are currently the main biomarkers of HRD and were frequently detected in our gastrointestinal and thoracic cancer cohort, often resulting in high SBS3 exposure and high HRD score. However, many tumors with phenotypic signatures consistent with HRD did not harbor *BRCA1/2* mutations. By surveying the whole genome and transcriptome, we were able to assess potential alternate mechanisms underlying the HRD signatures in these cases, and identified mutations and expression alterations that may contribute to these phenotypes. While methods have been employed to measure HRD-associated mutation signatures from cancer gene panels, whole-genome data improves sensitivity of HRD-associated signatures detection compared to exome or panel data^22,24^. Gene mutations and, as demonstrated in this paper, expression alterations that may contribute to HRD phenotypes will be missed if only using a limited gene panel. In particular, alterations in RAD51 family members including the homozygous loss of *XRCC2* and *RAD51B, BARD1*, and high expression of *RECQL5* were notable findings that may contribute to the high HRD scores, and SBS3 signatures in these *BRCA1/2* wild-type tumors. Increased levels of *RECQL5*, which is thought to cause HRD, was significantly associated with high HRD score in thoracic malignancies in our cohort, and has been described in several cancers including bladder and breast cancers^44,48–50^. This illustrates the utility in sequencing the whole genome and transcriptome for discovery of novel or emerging mechanisms of HRD, and may increase the ability to identify patients that may respond favorably to platinum therapy.

Limitations of this study include the heterogeneity of the GI and thoracic cohorts, encompassing various primary sites. Given that a goal of this study was to examine the relationship between HRD and platinum-based clinical outcomes, we sought to increase the sample size by including all patients in these two disease sites where HRD is an emerging biomarker despite the differences in histologies (including EGFR or ALK mutation status in lung cancer) and treatment histories. There is also heterogeneity in the type and timing of platinum therapy, and mechanistic and mutagenic differences have been reported between different platinum agents, such as oxaliplatin compared to cisplatin^51,52^. In this analysis, we made the decision to group platinum agents together as different agents are approved based on the tumor type and histology. Patients underwent fresh tumor biopsies for this study at different timepoints during their treatment course. Treatments can produce similar genomic scars (in particular microhomology deletions) to HRD, but platinum treatment prior to biopsy does not appear to induce HRD signatures since no significant correlation was found between platinum treatment duration prior to sequencing and SBS3 or HRD score (Supplementary Fig. 1C, D). Another potential confounder involves the chromosomal instability (CIN) subtypes that have been identified in gastric and colorectal cancers^53,54^. These CIN subtypes may be responsible for driving mechanisms of genomic instability independent of HRD that are not yet well characterized. We also recognize that there are inherent limitations with the OS analysis due to the advanced nature of this cohort, limited sample size affecting power of the study, as well as confounders related to the use of targeted therapies in the thoracic cohort. Furthermore, eligibility for the POG trial included an adequate ECOG performance status for later-line therapies, thus potentially introducing a selection bias.

While we were limited in sample size, our data indicate HRDetect that combines multiple genomic signatures may be more specific for identification of *BRCA1/2*-deficient tumors and platinum treatment duration compared to each signature individually, particularly in GI tumors. Widespread incorporation of mutational signature analysis in genomic profiling studies has shed light into the mutational processes in cancer, however the underlying mechanisms that drive the majority of signatures remain elusive. Distinct HRD-associated signatures emerge as a result of diverse compensatory pathways that are active when classical HR repair is defective; the weights of each respective distinct signature can vary depending on which gene or stage of the pathway is affected^14,30,55^. In contrast to classifiers that do not report individual signature metrics, aggregate signature methods like HRDetect that retain information pertaining to individual signature components enable discovery of novel gene signature associations.

Challenges associated with HRD-associated mutation signature detection must be addressed before mutation signature analysis may be widely used for clinical management. One such challenge relates to the complete and accurate assessment of structural variation across the genome. Inherent biases in the types of structural variants called by different methods and the limitations associated with short read sequencing that may not capture the full repertoire of structural variation present challenges for reproducibility and sensitivity. Long read sequencing promises increased sensitivity, particularly of complex structural rearrangements, and may improve sensitivity of HRD-associated SV signature detection^56^. Interpretation of HRD signature results for clinical care also presents a significant challenge. As accessibility to larger WGS datasets with matched treatment and response data across diverse tumor types as described in the POG study increases, measurement of HRD phenotypes and their association with clinical outcomes will be possible and will guide interpretation of mutation signature phenotypes^47,57^. Finally, given that WGS is best suited to achieve the highest accuracy and sensitivity of HRD signature detection compared to smaller panels, barriers to routine clinical implementation of WGS-based testing in the clinic remains a current limitation. However, great progress has been made in delivering WGS for clinical cancer care, and will continue to integrate into standard clinical practice as costs continue to fall^57–59^.

With the recognition of HRD as a predictive biomarker, HRD status, typically based on the presence of a number of mutations, is now being incorporated into prospective clinical trials. For instance, in the SWOG S1513 trial, this phase II study of second-line FOLFIRI with veliparib was stratified into three different HRD pancreatic cancer groups: (1) *BRCA1/2*; (2) non-BRCA core HRD including *ATM*, *ATR*, *PALB2*, *CDK12*, *RAD51C/D*, *BARD1*, *BRIP1* alterations; and (3) non-core HRD including *BLM*, *FANC*, *CHK1*, *CHK2*, *SLX4*, *ERCC*, *RIF1* alterations^9^. The presence of HRD is typically identified by commercially available panels, rather than by WGS. Our data in GI tumors suggest that SBS3 may represent another mechanism apart from established HRD mutations that may indicate HRD and susceptibility to platinum therapy. A similar finding was reported by Aguirre and colleagues in a study of prospective molecular annotation of patients with pancreatic ductal adenocarcinoma, where 5 of 71 patients also demonstrated a mutational SBS3 signature with no associated HRD mutations^13^. Prospective validation in a larger cohort of patients with GI malignancies is warranted to better comprehend this mechanism. A similar association was not seen in the cohort of patients with thoracic malignancies. In the thoracic cancer cohort, OS was shorter in the 7% of patients with *BRCA1/2* mutations. This may be related to the 19% of driver mutations (*ALK*, *EGFR*) present in the 12 of 63 non-*BRCA* patients, which likely contributed to the extended survival of this subgroup. Furthermore, patients included in this cohort received treatment before immune checkpoint blockade became standard of care in lung cancer for all patients with a PD-L1 status of ≥1%^60,61^. Therefore, platinum-based chemotherapy still represented the treatment backbone for a significant portion of patients and remained a later-line option for those who received upfront immune checkpoint blockade.

In conclusion, using a cohort of patients with GI and thoracic malignancies who underwent WGS and RNA-Seq, mutational SBS3 and HRDetect were more strongly associated with time to progression on platinum therapy compared to HRD score and CHORD in patients with GI malignancies. Evidence of alternate mechanisms of HRD including small mutations in *BARD1*, homozygous loss of RAD51 paralogs and overexpression of *RECQL5* indicate the complex nature of HRD, and illustrate the benefit of comprehensive genomic profiling to identify HRD patients. These data highlight potential predictive implications of phenotypic HRD profiling to complement somatic and germline mutation testing for the identification of patients who may benefit from exposure to platinum therapy.

## Methods

### Personalized OncoGenomics (POG) trial

In British Columbia, Canada, the Personalized OncoGenomics (POG) program is a translational research study that applies WGS and transcriptome analysis to guide treatment decision-making for patients with advanced malignancies, while leveraging the expertise of a multidisciplinary team of oncologists, pathologists, computational biologists, and bioinformaticians (NCT02155621)^47,62^. Fresh tumor biopsies and blood samples undergo WGS (80X tumor; 40X matched normal on Illumina HiSeq platform (San Diego, California) with 125 or 150 bp paired-end reads) and RNA sequencing (200 million reads, on Illumina HiSeq2500 or NextSeq500 with 75 bp paired-end reads). Sequence reads were aligned to the human reference genome (hg19) by the BWA tool and somatic single-nucleotide variants (SNVs), and small insertions and deletions (indels) were identified using SAMtools (v0.1.17) and Strelka (v1.0.6)^63–65^. Regions of copy number alteration (CNA) and losses of heterozygosity (LOH) were determined using CNAseq (v0.0.6) and APOLLOH (v0.1.1), respectively^66,67^. Structural variants (SVs) in RNA-seq data were detected by ABySS v1.3.4 and TransABySS (v1.4.10), Chimerascan (v0.4.5) and DeFUSE (v0.6.2); SVs in the DNA were identified using ABySS and TransABySS followed by Manta v1.0.0 and Delly v0.7.3^68–73^. SV calls from multiple algorithms were merged into a consensus caller MAVIS (v2.1.1) that performs a subsequent validation by local assembly^74^. WGS and transcriptome sequencing, with bespoke bioinformatics tools and analytic pipelines, helps to inform the molecular pathogenesis of tumors and potential therapeutic targets that are discussed at a molecular tumor board. Pathogenic and likely pathogenic germline variants in 98 known cancer predisposition genes (Supplementary Table 3) were analyzed according to the following guidelines. Germline SNVs and indels were identified in normal blood genomes using samtools (v0.1.17), annotated using SNPEff v4.1, population minor allele frequencies derived from the 1000 genomes v.1000g2015aug, and pathogenicity annotated using ClinVar v.20180905^64,75–77^. All coding and splice-site germline variants in 98 cancer predisposition genes were classified according to the American College of Medical Genetics 2015 guidelines using InterVar for partially automated classification followed by manual review^78,79^. This work was approved by and conducted under the University of British Columbia - BC Cancer Research Ethics Board (H12-00137, H14-00681). Written informed consent was obtained from all patients.

### Statistical analyses

We reviewed the WGS and RNA-Seq data among patients with metastatic GI and thoracic primaries between 2012–2018. The HRD score was calculated as the sum of loss of heterozygosity, telomeric allelic imbalance, and large-scale state transitions scores^26^. HRD was defined as a score ≥34 based on the fitting of the trimodal distribution of HRD score in thoracic and gastrointestinal cancers. The contribution of previously reported mutational signatures in the Catalogue of Somatic Mutations in Cancer (v3.1) was calculated using non-negative least squares optimization and high SBS3 exposure was defined as >0.05 NNLS based on the best separation of *BRCA1/2* mutated and non-mutated samples in a pan-cancer POG cohort^21^. HRDetect scores were computed using a logistic regression model with the same intercept and coefficients as those reported in the previously trained model, without adjustment^24^ The intercept was −3.364 and the coefficients were 1.611, 0.091, 1.153, 0.847, 0.667, and 2.398, respectively, for the six HRD signatures: (i) SBS3, (ii) SBS8, (iii) SV signature 3, (iv) SV signature 5, (v) the HRD index, and (vi) the fraction of deletions with microhomology. Somatic SNVs called by Strelka were used for single base substitution signature calculation. The contribution of previously reported mutational signatures in the Catalogue of Somatic Mutations in Cancer (COSMIC v3.1, https://cancer.sanger.ac.uk/cosmic/signatures) was calculated using Monte Carlo Markov Chain (MCMC) sampling (https://github.com/eyzhao/SignIT). MAVIS calls that were detected by more than one tool and for which the contig could be assembled were included in the analysis and the contribution of the previously reported SV mutational signatures was calculated using MCMC sampling (https://github.com/eyzhao/SignIT)^55^. The HRD index was computed as the arithmetic sum of loss of heterozygosity, telomeric allelic imbalance, and large-scale state transitions scores. The microhomology fraction was determined as the proportion of deletions which were larger than three base pairs and demonstrated overlapping microhomology at the breakpoints^26^. All signatures were log transformed and normalized so that each feature had a mean of 0 and standard deviation of 1^24^.

### HR genes

The expression of the following HR genes, selected based on their established roles in homologous recombination repair, were investigated to examine associations with high HRD score or high SBS3 exposure: *BARD1, BLM, BRCA1, BRCA2, BRIP1, DNA2, EXO1, MRE11A, NBN, PALB2, RAD50, RAD51, RAD51B, RAD51C, RAD51D, RAD52, RAD54L, RBBP8, WRN, XRCC2, XRCC3, ATM, BAP1, CUL3, EME1, ERCC1, ERCC4, FBXO18, GEN1, HELQ, MUS81, PARPBP, PCNA, POLD1, POLK, POLN, PSIP1, RAD51AP1, RECQL5, RIF1, RMI1, RMI2, RPA1, RPA2, RPA3, RTEL1, SLX1A, SLX4, TOP3A, TP53BP1*, and *USP11*.

### Survival analyses

Retrospective chart review was conducted to extract treatment and survival outcomes. Descriptive statistics were calculated to characterize the patient cohort. Overall survival (OS) was measured from initiation of first-line systemic therapy to date of death or last follow-up.

Time to progression on platinum therapy (TTPp) was calculated from initiation of platinum agent to end of platinum treatment or start of next-line treatment if treatment end date was not available. Cox regression analyses were conducted to examine the association between HRD and TTPp. All tests were two-sided, with *p* < 0.05 as the cutoff for statistical significance. Stata version 15.1 was used for all statistical analyses (College Station, Texas, USA).

### Reporting summary

Further information on research design is available in the Nature Research Reporting Summary linked to this article.

## Supplementary information

Supplementary Tables and Figures

REPORTING SUMMARY

## Data Availability

Genomic data generated within the Personalized OncoGenomics study are actively submitted to the European Genome-phenome Archive (EGA) under accession number #EGAS00001001159.

## Code availability

The bioinformatics analyses were performed using open-source software, including Burrows–Wheeler alignment tool (v.0.5.7 for up to 125 bp reads and v.0.7.6a for 150 bp reads)^63^, CNAseq^66^ (v.0.0.6), APOLLOH^67^ (v.0.1.1), SAMtools^64^ (v.0.1.17), Strelka^65^ (v.1.0.6), SNPEff^75^ (v.3.2 for somatic and v.4.1 for germline), ABySS^68^ (v.1.3.4), TransABySS^73^ (v.1.4.10), Chimerascan^69^ (v.0.4.5), DeFuse^70^ (v.0.6.2), Manta^73^ (v.1.0.0), Delly^71^ (v.0.7.3), MAVIS^74^ (v.2.1.1), HRDtools^26^ (v.0.0.0.9), SignIT (https://github.com/eyzhao/SignIT), ClinVar^80^ (v.20180905), InterVar^79^. Additional packages used for meta-analyses include Python packages pandas (v.1.0.1), numpy (v1.18.1) and scipy (v.1.4.1). Additional processing involved in-house scripts that are available upon request.
