# Supplementary material for: Homologous recombination deficiency signatures in gastrointestinal and thoracic cancers correlate with platinum therapy duration
Source: NPJ Precis Oncol. 2023 Mar 24;7:31. doi: 10.1038/s41698-023-00368-x (PMC10039042; doi:10.1038/s41698-023-00368-x)
Supplement: Supplementary file 1 — Supplementary Tables and Figures [file 41698_2023_368_MOESM1_ESM.docx]

**Supplemental Figure 1.** SBS3 exposures with the threshold of 0.05 (dashed line) (A) and microhomology deletions (B) in thoracic patients with and without smoking signature, SBS4.

SBS3 exposures (C) and HRD score (D) in tumours with previous platinum treatment (median days = 106). Box plots represent the median (black line), upper (75th) and lower (25th) quartiles of the distribution and whiskers represent the limits of the distribution (1.5-times interquartile range). All p-values are calculated by two-sided Wilcoxon rank sum tests, comparing the treated and non-treated samples, with no correction.


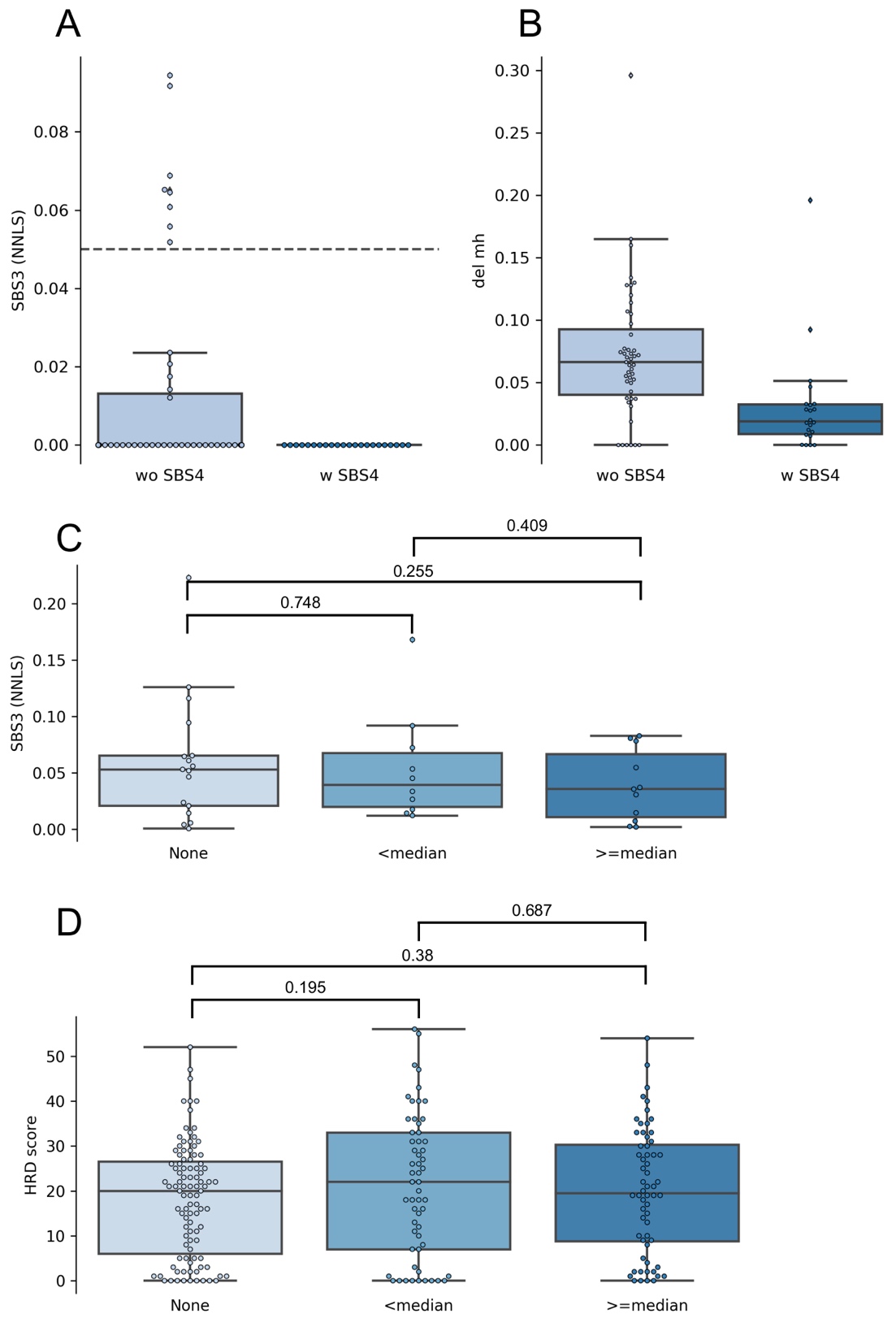


**Supplemental Figure 2.** *RECQL5* expression in patients with low (<0.05) and high SBS3 (>=0.05) scores (A) and in patients with or without RECQL5 copy gains (B) in the combined and individual GI and thoracic (THR) cohorts. All p-values presented on the boxplots are determined by Wilcoxon rank sum test (Holm-Bonferroni correction). Box plots represent the median (black line), upper (75th) and lower (25th) quartiles of the distribution and whiskers represent the limits of the distribution (1.5-times interquartile range).


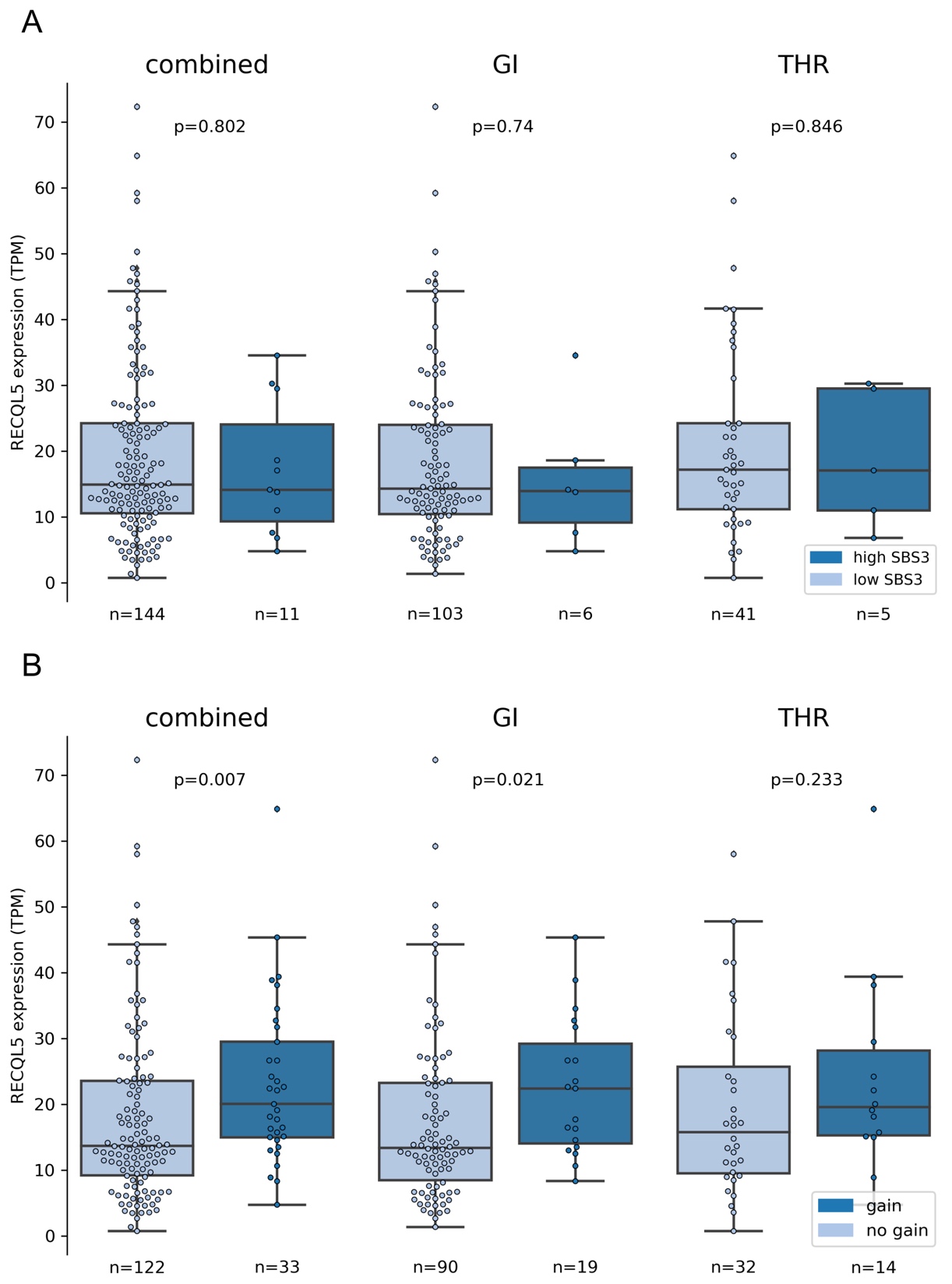


**Supplemental Figure 3.** Kaplan-Meier overall survival curves for patients with metastatic gastrointestinal malignancies by HRD score (A), SBS3 exposure (B), and BRCA status (C)


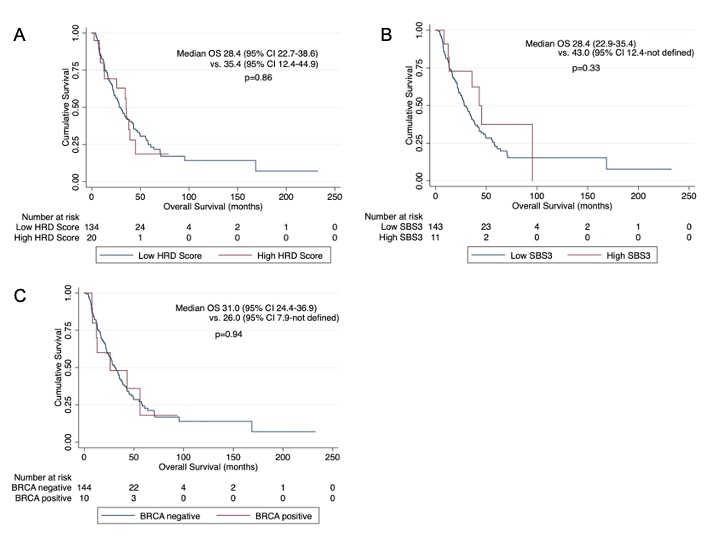


S**upplemental Figure 4.** Kaplan-Meier overall survival curves for patients with metastatic thoracic malignancies by HRD score (A), SBS3 exposure (B), and BRCA status (C)


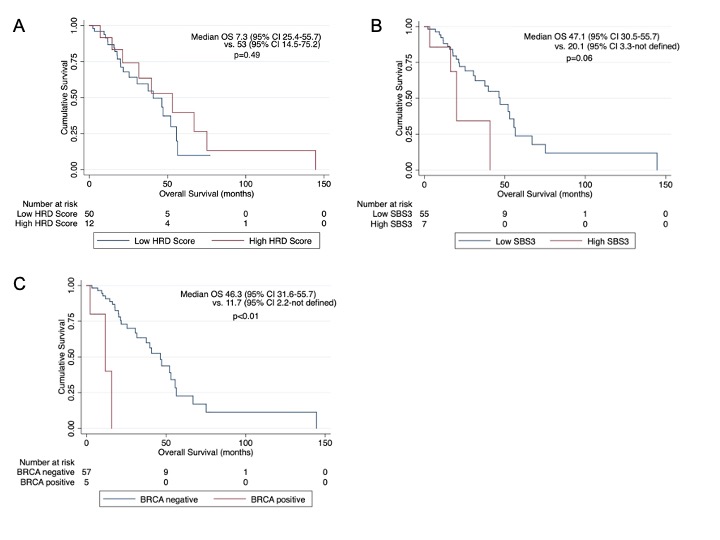


**Supplemental Table 1. A)** Pathogenic *BRCA1/2* germline alterations in GI and THR cohorts; B) *BRCA1/2* germline variants with unknown significance in GI and THR cohorts; C) Benign and likely benign *BRCA1/2* germline alterations in GI and THR cohorts. One of the patients (*) had multiple benign mutations.

A)

| **cohort** | **gene** | **position** | **HGVS- cDNA** | **HGVS- protein** | **dbsnp** |
| --- | --- | --- | --- | --- | --- |
| GI | BRCA1 | 41246697 | c.844_850dup | p.Gln284Leufs |  |
| GI | BRCA1 | 41276044 | c.68_69delAG | p.Glu23Valfs |  |
| GI | BRCA1 | 41217006 | exons 1-17 deletion | |  |
| GI | BRCA2 | 32914766 | c.6275_6276delTT | p.Leu2092fs | rs11571658 |
| GI | BRCA2 | 32912337 | c.3847_3848delGT | p.Val1283Lysfs |  |
| GI | BRCA2 | 32936732 | c.7878G>C | p.Trp2626Cys | rs80359013 |
| THR | BRCA2 | 32893238 | c.92G>A | p.Trp31Ter | rs397508045 |
| THR | BRCA2 | 32937507 | c.8168A>C | p.Asp2723Ala | rs41293513 |
| THR | BRCA2 | 32915053 | c.6566dupA | p.Asn2189fs |  |

B)

| **cohort** | **gene** | **position** | **HGVS- cDNA** | **HGVS- protein** | **dbsnp** |
| --- | --- | --- | --- | --- | --- |
| GI | BRCA2 | 32911557 | c.3065A>G | p.His1022Arg |  |
| GI | BRCA2 | 32914509 | c.6017G>C | p.Ser2006Thr | rs144784912 |

C)

| **cohort** | **gene** | **position** | **HGVS- cDNA** | **HGVS- protein** | **dbsnp** |
| --- | --- | --- | --- | --- | --- |
| GI | BRCA2 | 32914592 | c.6100C>T | p.R2034C | rs1799954 |
| GI | BRCA2 | 32930598 | c.7469T>C | p.Ile2490Thr | rs11571707 |
| GI | BRCA2 | 32972884 | c.10234A>G | p.Ile3412Val | rs1801426 |
| GI | BRCA2 | 32972884 | c.10234A>G | p.Ile3412Val | rs1801426 |
| GI | BRCA2 | 32930663 | c.7534C>T | p.Leu2512Phe | rs80358980 |
| GI | BRCA2 | 32914592 | c.6100C>T | p.Arg2034Cys | rs1799954 |
| GI | BRCA2 | 32912684 | c.4192G>C | p.Ala1398Pro |  |
| GI | BRCA2 | 32913070 | c.4578A>G | p.Thr1526= | rs202022822 |
| GI | BRCA2 | 32893344 | c.198G>A | p.Gln66= | rs28897700 |
| GI | BRCA1 | 41246130 | c.1418A>T | p.Asn473Ile | rs80357057 |
| GI | BRCA2 | 32914236 | c.5744C>T | p.Thr1915Met | rs4987117 |
| GI | BRCA2 | 32913106 | c.4614T>C | p.Ser1538= | rs45520945 |
| GI | BRCA1 | 41223048 | c.4883T>C | p.Met1628Thr | rs4986854 |
| THR | BRCA2 | 32913708 | c.5218_5223del | p.Leu1740_Ser1741del |  |
| THR | BRCA2 | 32914260 | c.5768A>C | p.Asp1923Ala | rs45491005 |
| THR* | BRCA2 | 32906890 | c.1275A>G | p.Glu425Glu | rs34355306 |
| THR* | BRCA2 | 32953879 | c.8954-5_8954-2del |  |  |
| THR* | BRCA1 | 41251893 | c.446A>C | p.Glu149Ala | rs397507233 |

**Supplemental Table 2.**

1. Cox regression time to progression on platinum analysis in GI cohort

|  | **Hazard ratio (95% CI)** | **p-value** |
| --- | --- | --- |
| **SBS3 (low vs. high)** | **0.35 (0.14-0.91)** | **0.031** |
| Tumor site  Pancreas vs. upper GI  Colorectal vs. upper GI  Other GI primary vs. upper GI | 0.99 (0.47-2.09)  0.81 (0.44-1.50)  1.12 (0.46-2.72) | 0.99  0.51  0.80 |
| Lines of platinum therapy | 0.51 (0.18-1.47) | 0.21 |

1. Cox regression overall survival analysis in GI cohort

|  | **Hazard ratio (95% CI)** | **p-value** |
| --- | --- | --- |
| **Duration of platinum-based therapy** | **0.98 (0.95-0.99)** | **0.02** |
| HRD score (low vs. high) | 1.19 (0.64-2.22) | 0.57 |
| BRCA mutation (no vs. yes) | 0.74 (0.33-1.63) | 0.45 |
| Tumor site  Pancreas vs. upper GI  Colorectal vs. upper GI  Other GI primary vs. upper GI | 1.04 (0.55-1.94)  0.35 (0.20-0.63)  0.27 (0.13-0.57) | 0.92  <0.01  <0.01 |

**Supplemental Table 3.** List of 98 known cancer predisposition genes

| APC  BMPR1A  SMAD4  BRCA1  BRCA2  CDKN2A  FLCN  MEN1  MLH1  MSH2  MSH6  PMS2  MUTYH  TP53  PTEN  SDHB  SDHD  SDHC  STK11  TERT  VHL  RET  SDHAF2  PRKAR1A  TMEM127  MAX  CHEK2  BAP1  FH  SMARCB1  CDH1  CDC73  GATA2  CDKN1B  ATM  DKC1  EPCAM  KIT  MET  PDGFRA  PTCH1  TERC  TINF2  WRN  RECQL4  AKT1  ALK  ATR  AXIN2  BARD1  BLM  BRIP1  CBL  CDK4  DICER1  EGFR  ERCC2  ERCC3  ERCC4  ERCC5  ETV6  EZH2  ABRAXAS  FANCA  FANCC  GREM1  HNF1A  HRAS  IDH1  MITF  MRE11  NBN  NF1  NF2  NSD1  PALB2  PAX5  PHOX2B  PIK3CA  PMS1  POLD1  POLE  PTPN11  RAD50  RAD51  RAD51B  RAD51C  RAD51D  RB1  RUNX1  SDHA  SH2D1A  SMARCA4  SUFU  TGFBR1  TSC1  TSC2  WT1 |
| --- |
